# Orexin neurons track temporal features of blood glucose in behaving mice

**DOI:** 10.1038/s41593-024-01648-w

**Published:** 2024-05-21

**Authors:** Paulius Viskaitis, Alexander L. Tesmer, Ziyu Liu, Mahesh M. Karnani, Myrtha Arnold, Dane Donegan, Eva Bracey, Nikola Grujic, Tommaso Patriarchi, Daria Peleg-Raibstein, Denis Burdakov

**Affiliations:** 1 Department of Health Sciences and Technology, Swiss Federal Institute of Technology (ETH Zürich), Zurich, Switzerland; 2 Department of Neurology, The First Affiliated Hospital of Xi’an Jiaotong University, Xi’an, China; 3 Centre for Discovery Brain Sciences, University of Edinburgh, Edinburgh, UK; 4 Institute of Pharmacology and Toxicology, University of Zurich, Zurich, Switzerland

**Keywords:** Neurophysiology, Sensory processing

## Abstract

Does the brain track how fast our blood glucose is changing? Knowing such a rate of change would enable the prediction of an upcoming state and a timelier response to this new state. Hypothalamic arousal-orchestrating hypocretin/orexin neurons (HONs) have been proposed to be glucose sensors, yet whether they track glucose concentration (proportional tracking) or rate of change (derivative tracking) is unknown. Using simultaneous recordings of HONs and blood glucose in behaving male mice, we found that maximal HON responses occur in considerable temporal anticipation (minutes) of glucose peaks due to derivative tracking. Analysis of >900 individual HONs revealed glucose tracking in most HONs (98%), with derivative and proportional trackers working in parallel, and many (65%) HONs multiplexed glucose and locomotion information. Finally, we found that HON activity is important for glucose-evoked locomotor suppression. These findings reveal a temporal dimension of brain glucose sensing and link neurobiological and algorithmic views of blood glucose perception in the brain’s arousal orchestrators.

## Abstract (web summary)

How the brain tracks blood glucose dynamics is unclear. Viskaitis et al. show that hypothalamic orexin/hypocretin cells track glucose concentration and rate of change and that their activity is important for glucose-evoked locomotor suppression.

## Main

Many sensory inputs to our brain change rapidly in time. Knowing the rate of change of an input, as opposed to just the current value, allows for the prediction of a future state and a timelier response to this new state. For key external sensory inputs, classical experiments have established that the brain tracks both their current value and rate of changes^1^. Whether the brain also tracks the rate of change of internal body variables, such as glucose concentration, is less well understood. Glucose is increasingly appreciated as an important internal variable that—separately from its ubiquitous role as a metabolic fuel—acts as a sensory input to the brain, detected by specialized ‘glucose-sensing’ neurons located only in certain brain areas, such as the lateral hypothalamus (LH)^2^.

In the LH, hypocretin/orexin neurons (HONs) are a cornerstone of adaptive arousal across species and have been proposed to be glucose sensors^3–7^. Many studies of HONs focused on disentangling their gene expression fingerprints, causal circuit interactions and long-term plasticity. An equally important aspect that is much less studied concerns the temporal relations between internal variables and HON activation. In particular, the fundamental question of whether HONs track the temporal features of blood glucose, such as the rate of change, has been difficult to resolve because prior studies did not simultaneously record HON activation and blood glucose dynamics at a sufficient temporal resolution. This is especially important because HON activity is thought to be regulated by multiple neural inputs and can be linked to metabolic rates and energy use^8–13^. This emerging multivariate context of HON regulation makes it unclear whether HON activity reflects blood glucose at all.

To address these gaps in knowledge, here we used LH-implanted optics in behaving male mice to record HON population activity or the separate responses of >900 individual HONs, with concurrent electrochemical monitoring of blood glucose dynamics and multiple physiological and behavioral variables. These parallel experimental measurements and their multivariate statistical analyses enabled us to probe the temporal features of blood glucose dynamics tracked by HONs in behaving mice. By documenting how HON loss- and gain-of-function manipulations affect behavioral responses to glucose, we also probed the causal role of HONs in behavioral responses to glucose. We found that maximal HON responses to blood glucose deviations occur in temporal anticipation of the glucose peaks due to rate-of-change (first derivative) tracking of glucose. At the single-cell resolution, we found ‘rate-of-change’ and ‘current-value’ glucose trackers operating in parallel, with each transmitting additional information about the animal’s locomotion state. Finally, our experiments revealed that HONs are important for glucose-mediated locomotor control.

## Results

### Blood glucose fluctuations rapidly affect HONs in vivo

**Fig. 1:**
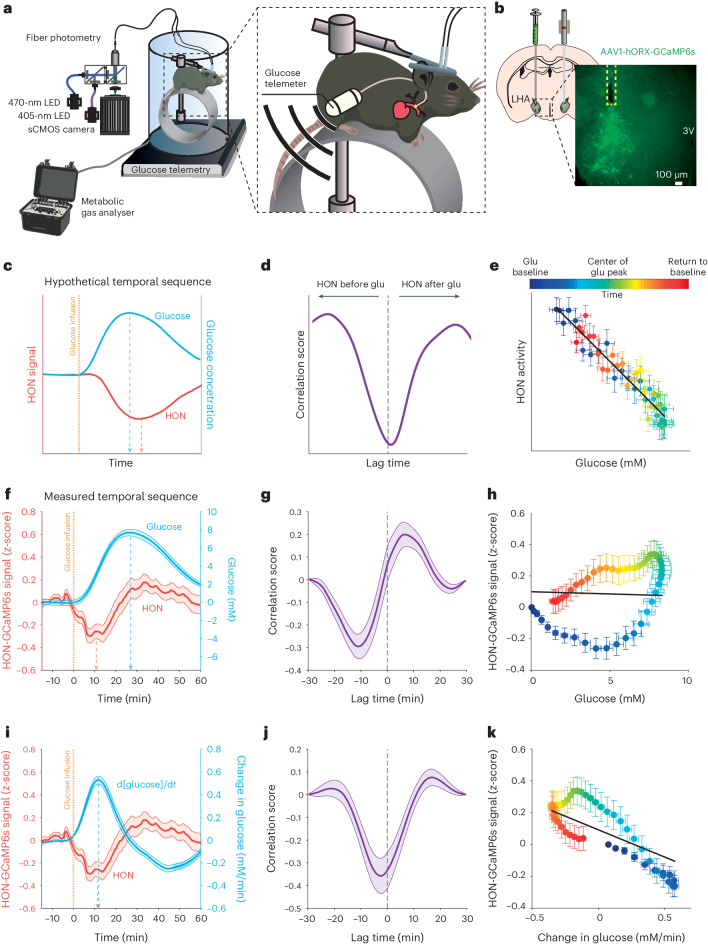
Temporal relations of HON population activity and blood glucose. **a**, Scheme of the experimental setup for simultaneous fiber photometry, indirect calorimetry, glucose and temperature telemetry, and locomotion recordings. sCMOS, scientific complementary metal–oxide–semiconductor; LED, light-emitting diode. **b**, Left: stereotaxic surgery schematic. LHA, lateral hypothalamic area. Right: expression of GCaMP6s in HONs for fiber photometry. The dashed square box indicates the fiber location. 3V, third ventricle. **c**–**e**, Hypothetical temporal relations of HON signal and blood glucose versus time (**c**), HON–glucose correlation versus lag time between their peaks (**d**) and HON activity versus glucose (**e**), Glu, glucose. **f**–**h**, Measured temporal relations of HON activity and blood glucose simultaneously recorded in the same experiments (the preinfusion glucose baseline was subtracted from the glucose values for visual clarity). **f**, HON activity and blood glucose concentration traces; the peak HON response preceded the peak glucose concentration. Glucose infusion significantly decreased the HON photometry signal (between 0 and 10 min) compared to baseline (−10 to 0 min), *P* < 0.0001, *t* = 4.146, degrees of freedom (d.f.) = 105. **g**, Cross-correlation of HON activity and blood glucose. **h**, The linear fit did not explain variability, and the fit slope was not different from zero: *R*^2^ = 0, *P* = 0.288. **i**–**k**, Measured temporal relations of HON activity and the glucose derivative (d[glucose]/d*t*). The linear fit explained some variability, and the slope was significantly nonzero (**i**): *R*^2^ = 0.04, *P* < 0.0001. In **f**–**k**, data are means and s.e.m. of *n* = 57 IG glucose infusion responses from 22 mice. Data are smoothed by a 10-min moving mean for visualization. Source data

To correlate HON dynamics with concurrently measured changes in the blood glucose concentration, we designed and implemented an experimental paradigm that combined telemetry of carotid electrochemical glucose sensors and fiber photometry of HON-selective GCaMP6s fluorescent neural activity sensors (Fig. 1a,b and Methods).

Previous models of HON glucose sensing predict that HON inhibition should proportionally track the absolute glucose concentration, with a sensing delay of a few minutes^6,7^. If such concentration-proportional sensing occurred in the intact organism whose blood and brain glucose levels rapidly equilibrate^14^, we would expect maximal HON inhibition to occur after the glucose peak (Fig. 1c,d), and a negative monotonic relation between HON activity and glucose concentration (Fig. 1e).

**Extended Data Fig. 1:**
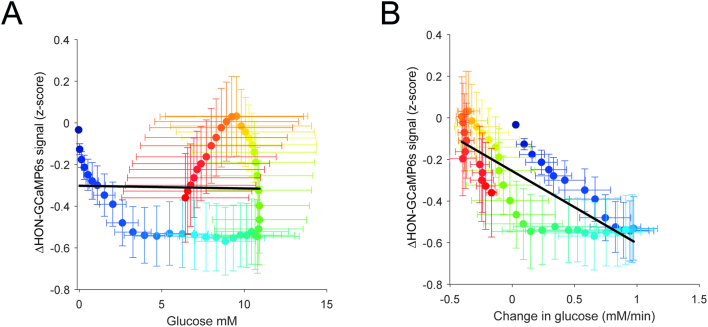
Relationship between blood glucose and HON activity after IP injection of glucose. Related to Fig. 1. **(A)** HON activity did not have a linear relationship with varying blood glucose after intraperitoneal (IP) injection of glucose. Linear fit did not explain variability and fit slope was not different from zero: R2 = 0.0005, p = 0.87. **(B)** There was a significant linear relationship of HON activity with changes in blood glucose and the slope was significantly non-zero: R2 = 0.64, p < 0.0001. The slope of the relation between HON activity and change in blood glucose was not affected by the route of glucose administration: IG (Fig. 1k) vs IP (this figure, panel B); F test, p = 0.17. IP glucose responses quantified using 19 HON responses from 8 mice & 4 glucose recordings in 4 mice. Data are presented as means and s.e.m. Source data

We found that, in vivo, an increase in blood glucose rapidly and significantly inhibited HON population activity (Fig. 1f). However, the timing of HON inhibition strikingly diverged from a time-delayed copy of the blood glucose waveform. Instead, the HON inhibitory response was seen only during blood glucose increase, with the peak HON response occurring several minutes before—rather than after—the blood glucose peak (Fig. 1f,g). Also, unexpectedly, HON activity subsequently returned to control levels when glucose was at the peak level and stable and then increased when the glucose level was declining (Fig. 1f,g). As a result, the HON activity state, as a function of blood glucose, displayed a hysteresis profile (Fig. 1h) rather than the expected monotonic relationship (Fig. 1e). From this noncanonical temporal profile of the HON glucose response, we hypothesized that HON inhibition tracked the rate of change of glucose (that is, its first derivative, d[glucose]/d*t*). Indeed, replotting of differentiated glucose data revealed that the dynamics of HON inhibition was a close mirror image of the glucose derivative (Fig. 1i,j), and there was a significantly negative linear relationship between HONs and the glucose derivative (Fig. 1k). This relationship was similar irrespective of the glucose administration route (intragastric (IG; Fig. 1) or intraperitoneal (IP; Extended Data Fig. 1)).

Together, these data show that the HON population is rapidly inhibited by an increase in blood glucose, and this inhibition tracks the rate of change of blood glucose.

### Encoding of multiple variables in HON low-frequency waves

Blood glucose fluctuations are expected to affect multiple behavioral and metabolic parameters, and HON activity may covary with a number of those^15,16^. This raises a fundamental question: do HONs specialize in glucose sensing, or is the influence of glucose relatively minor compared to that of other variables that may affect HONs in vivo?

**Fig. 2:**
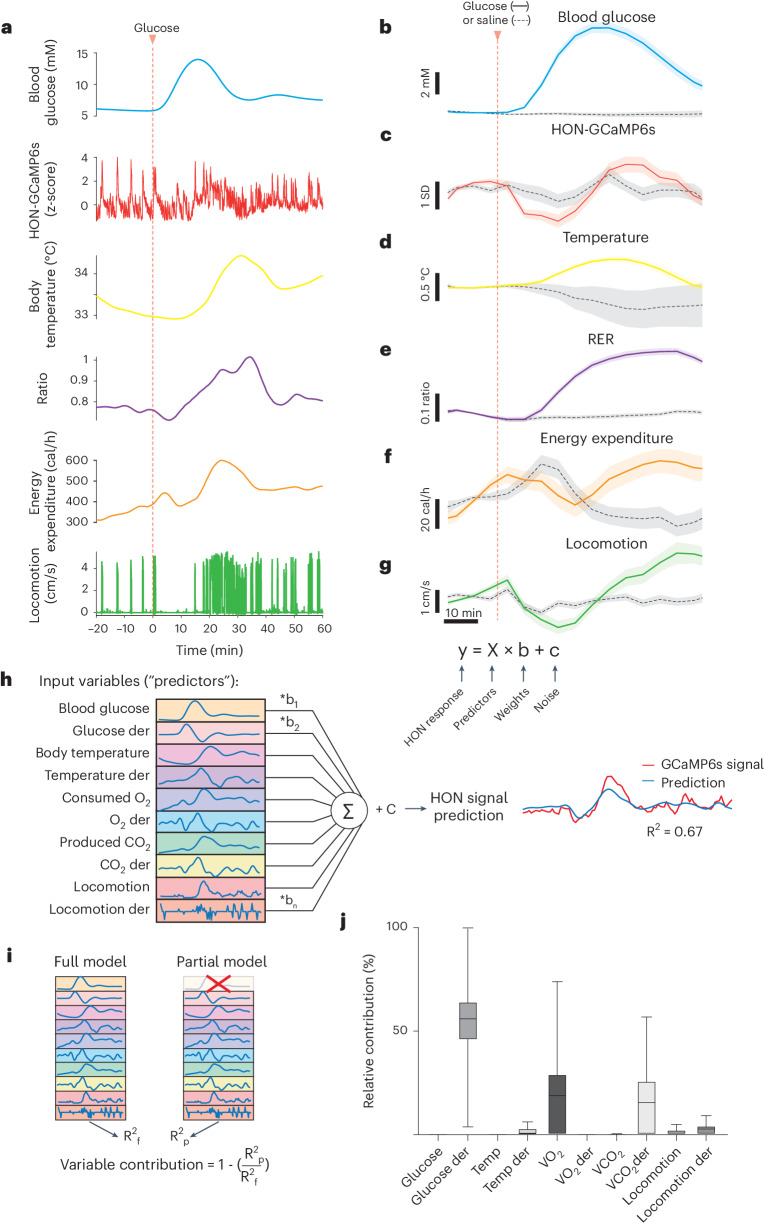
Relative influence of behavioral and metabolic variables on HONs. **a**, Example multiparameter data from a single recording session. **b**–**g**, Group data for the experiment shown in **a**. Data are shown in 5-min bins for visualization only. The 5- to 25-min postinfusion was used for statistics. Compared to saline infusion, glucose infusion increased the blood glucose level (**b**) (*t*-test *P* < 0.0001; d.f. = 26, *t* statistics = −5.193; *n* = 7 and 21 responses from 6 and 6 animals to saline and glucose, respectively), reduced HON activity responses (**c**) (*t*-test *P* = 0.023; d.f. = 69, *t* statistics = 2.321; *n* = 39 and 32 responses from 13 and 10 animals to saline and glucose, respectively), increased body temperature (**d**) (*t*-test *P* = 0.038; d.f. = 31, *t* statistics = −2.167; *n* = 7 and 26 responses from 7 and 8 animals to saline and glucose, respectively), increased the respiratory exchange ratio (RER; **e**) (*t*-test *P* < 0.0001; d.f. = 89, *t* statistics = −5.03; *n* = 59 and 32 responses from 30 and 15 animals to saline and glucose, respectively), did not alter energy expenditure (**f**) (*t*-test *P* = 0.10; d.f. = 89, *t* statistics = 1.648; *n* = 59 and 32 responses from 30 and 15 animals to saline and glucose, respectively) and reduced running (**g**) (*t*-test *P* < 0.0001; d.f. = 117, *t* statistics = 4.39; *n* = 80 and 39 responses from 32 and 19 animals to saline and glucose, respectively). The 10-min time bar shown in **g** also applies to **a**–**f**. Data in **b**–**g** are means and s.e.m. **h**, Depiction of the workflow to predict HON activity from the simultaneously measured set of physiological variables. der, derivative. **i**, Illustration of the testing of the contributions of input variables. **j**, Ranking of input variable contributions for accuracy in modeling HON activity after glucose infusion (box-and-whisker plot: center line, median; box, 25th–75th percentiles; whiskers, minimum–maximum extremes). Data are from 17 recording sessions in 5 mice. Source data

Answering this question requires simultaneous experimental tracking of multiple physiological variables and a method to quantify how much HON activity variability is attributed to each separate variable despite the presence of multiple variables. To achieve this, we designed an experimental and analysis workflow enabling the comonitoring of multiple variables at the same temporal resolution, including blood glucose, CO_2_ and O_2_ respiratory gas exchange, HON-GCaMP6s activity, body temperature and locomotion (Figs. 1a and 2a). We then used the resulting data matrix (Fig. 2a–g) to quantitatively predict the HON population activity based on the other physiological variables (‘predictors’) with a multiple linear regression encoding model^17^ (Fig. 2h and Methods). Predictors also included the first derivatives of the variables (Fig. 2h) because this temporal feature was implicated in HON responses to glucose (Fig. 1i–k).

**Extended Data Fig. 2:**
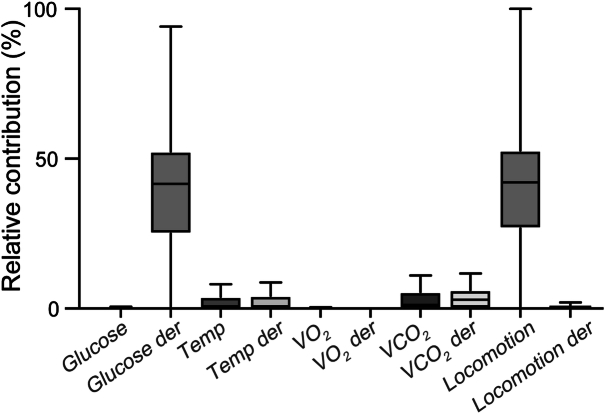
Glucose derivative is a top predictor across a wide range of glucose infusion parameters. Related to Fig. 2. Unlike the dataset than shown in Fig. 2 (0.24 g/ml, 90 μl/min for 10 min), here a wider range of the infused glucose parameters were used: glucose concentration was either 0.12, 0.146 or 0.45 g/ml, the infusion rate was varied between 66.7, 90 and 180 μl/min, and duration was either 2.5, 10 or 15 min. N = 11 sessions from 5 mice. (box and whisker plot, showing median, 25th to 75th percentile box, and min/max extremes).

Using this encoding model, we calculated the relative contribution of each physiological variable to the slow HON population response by quantifying how much of the explained variance decreased when that variable was removed from the model (Fig. 2i). The highest relative contribution was attributed to the derivative of glucose (29.7 ± 2% relative contribution to the explained variance), followed by consumed oxygen volume (VO_2_, 21.1 ± 3.6%) and the derivative of produced carbon dioxide volume (VCO_2_, 17.6 ± 2.8%) (Fig. 2j; confirmed for a variety of glucose infusion parameters in Extended Data Fig. 2).

Thus, when the covariation of HON activity across multiple physicochemical and behavioral factors is considered, the glucose derivative emerges as a strong determinant of information transmitted by the HON population.

### Multiplexed tracking of glucose and running in single HONs

**Fig. 3:**
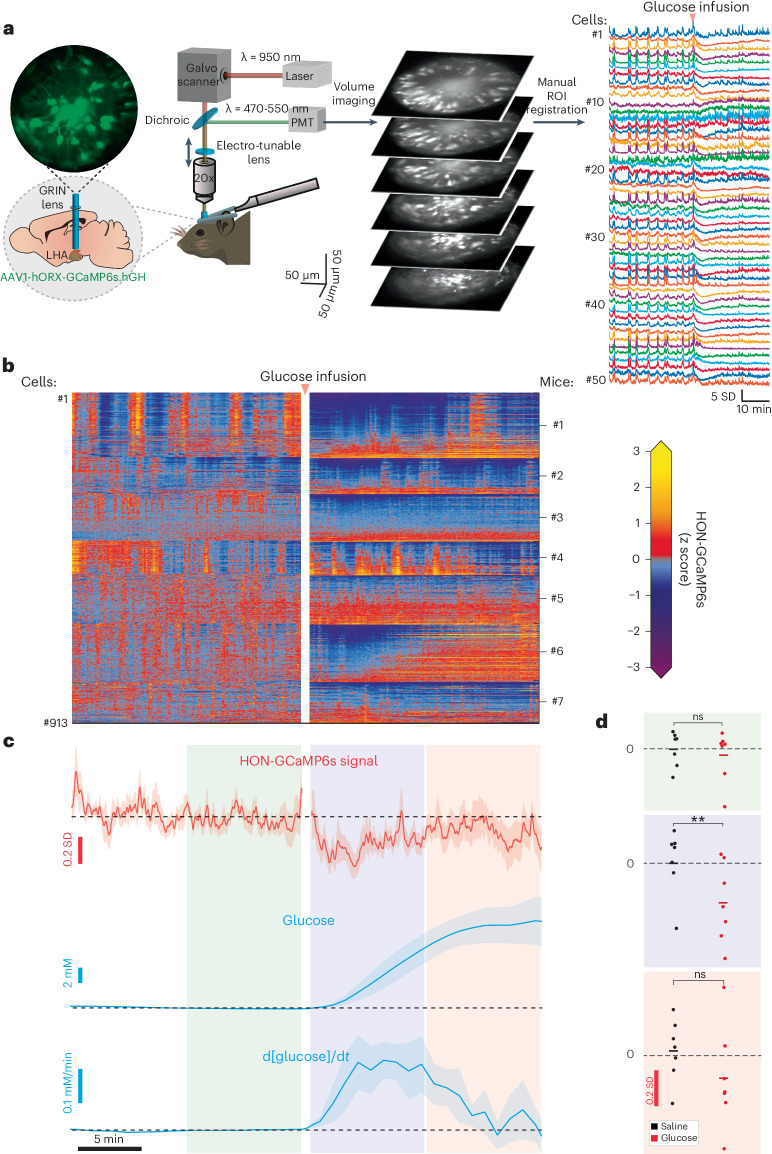
Single-cell resolution analysis of HON responses to blood glucose fluctuations. **a**, Scheme of two-photon imaging of individual HONs in behaving mice. ROI, region of interest. **b**, Cellular responses of 913 HONs from 7 mice to glucose infusion (data for the saline control are shown in Extended Data Fig. 3). **c**, Temporal alignment of average HON responses (top) to blood glucose concentration (middle) and its derivative (bottom). Glucose infusion arrow shown in **b** also applies to **c**, and time bar shown in **c** also applies to **b**. **d**, HONs were significantly inhibited only during the high-glucose-derivative period (2–11 min, blue box) but not during the high-absolute-glucose period (11–20 min, red box) or the baseline period (−11 to −2 min, green box). Two-way repeated-measures (RM) analysis of variance (ANOVA): treatment *P* = 0.029, *F*(1.0, 6.0) = 8.216; Šidák multiple-comparison correction: *P* = 0.709 (NS) (baseline period), **P = 0.005 (high-glucose-derivative period), *P* = 0.534 (NS) (high-absolute-glucose period), *n* = 7 mice. NS, not significant. Data are shown as means and individual points (*n* = 7 mice). Breaks in the neural activity recordings are due to the laser shutter being closed during infusions in most of the experiments. Data are presented as means and s.e.m. Source data

We next sought to determine whether blood glucose homogeneously affects individual HONs. Therefore, we switched our HON activity recording mode from bulk photometry to a single-cell resolution using two-photon volumetric imaging of HONs through hypothalamus-implanted gradient index (GRIN) lenses^15^ (Fig. 3a). We confirmed that, at the population level, HON glucose responses were similar across the two recording modes, by observing comparable glucose responses in fiber photometry (above) and summed two-photon HON imaging (Fig. 3b–d).

**Fig. 4:**
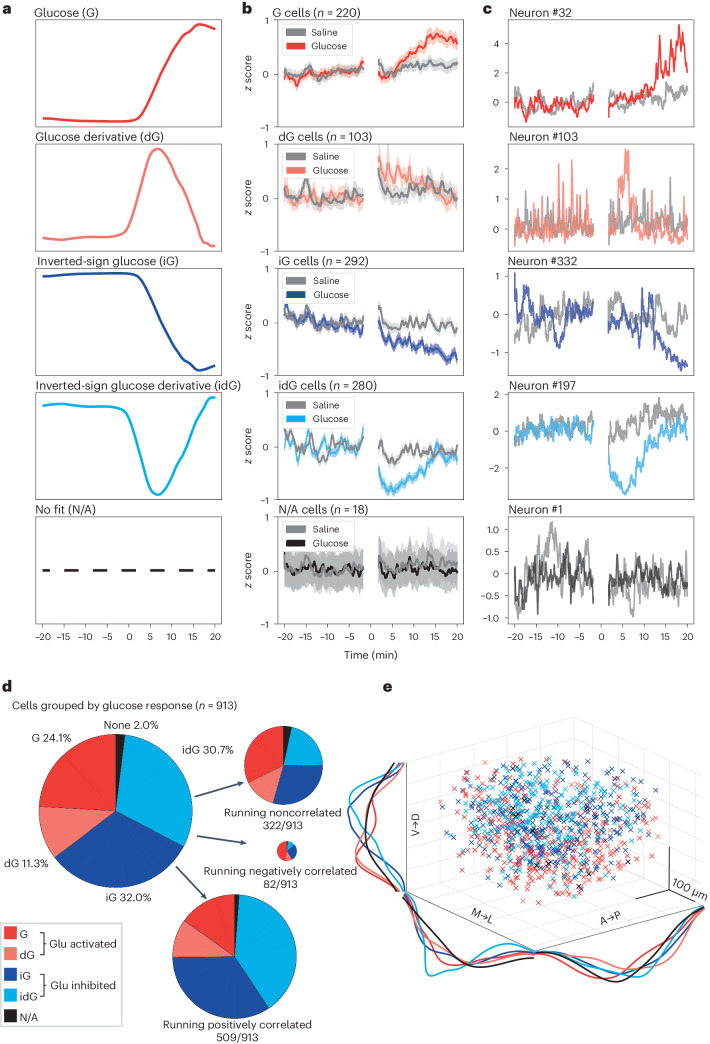
Classification of single-cell HON responses to blood glucose and running. **a**, Fitting templates based on the recorded blood glucose dynamics: glucose (G), glucose derivative (dG), inverted glucose (iG), inverted glucose derivative (idG) and no fit (N/A). **b**, Average HON activity traces for each of the glucose-response classes. **c**, Examples of individual neurons from each class. The *y*-axis label in **b** also applies to **c**. **d**, Relative proportions of HON subsets with regard to their responses to glucose and further subdivided by correlations with running. **e**, Anatomical distribution of HON subsets. V, ventral; D, dorsal; M, medial; L, lateral; A, anterior; P, posterior. Breaks in the neural recordings are due to the laser shutter being closed during infusions in most of the experiments. Data are presented as means and s.e.m.

**Extended Data Fig. 3:**
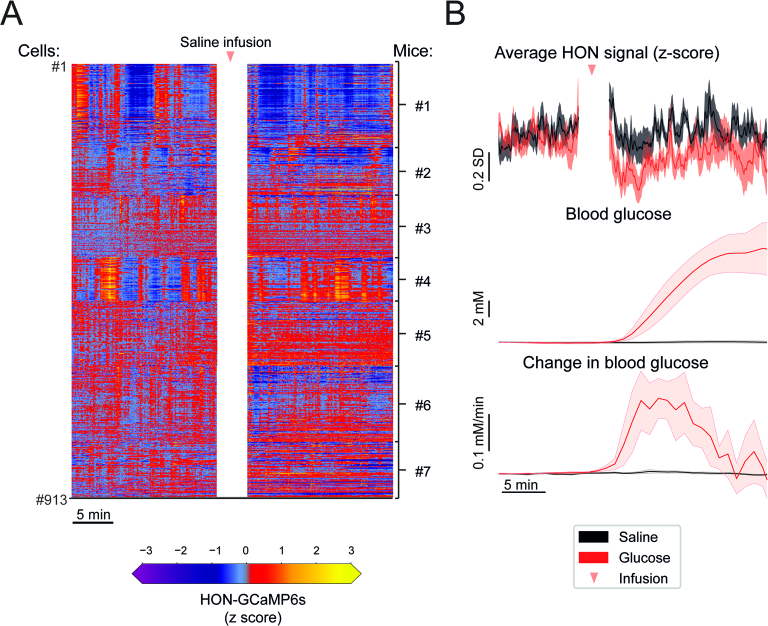
Single cell HON responses to saline and glucose infusions. Related to Fig. 3. **(A)** Individual responses of 913 HONs to saline infusion from 7 mice. **(B)** Temporal alignment of average HON responses (top) to blood glucose concentration (middle) and its derivative (bottom) after saline or glucose infusions. Blood glucose data is from the same recording sessions as shown in Extended Data Fig. 1. Breaks in the neural recordings are due to laser shutter being closed during infusions in majority of the experiments. Data are presented as means and s.e.m.

**Extended Data Fig. 4:**
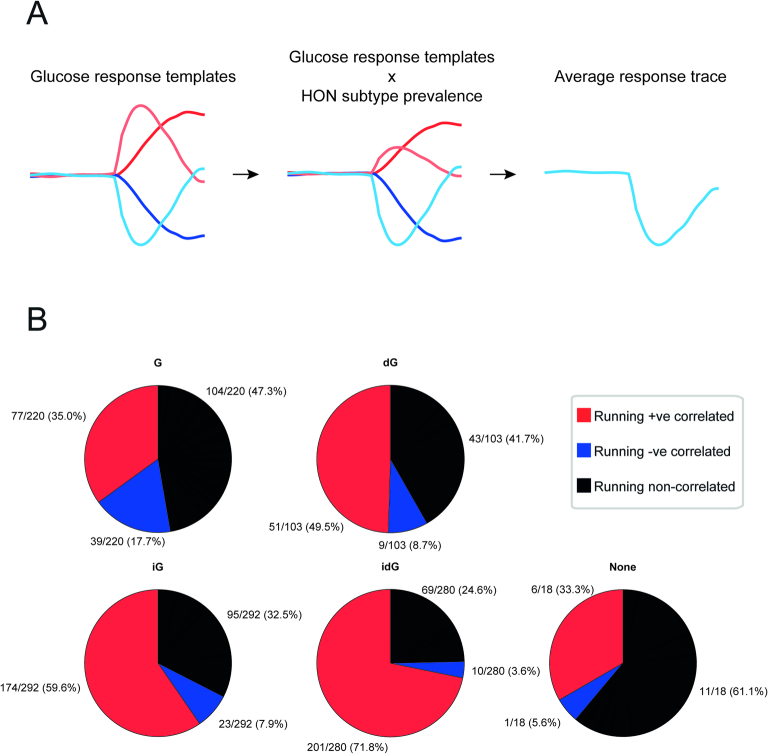
HON population response to glucose and correlation of single cell HON responses to glucose infusions versus running. Related to Fig. 4. **(A)** Using blood glucose templates from Fig. 4A and multiplying them by the relative prevalence of individual HONs that fit these profiles, we obtained an average response trace that resembles that of the actual HON population response after the infusion of glucose. **(B)** Glucose inhibited cells (iG and idG) tended to also have a larger proportion of +ve correlated running cells.

In each mouse (*n* = 7), we simultaneously resolved the activity of 92–180 HONs. We analyzed a total of 913 HONs from 7 mice (the full dataset is shown in Fig. 3b for glucose and Extended Data Fig. 3 for vehicle control). To compare the glucose responses of individual cells, we fitted each cell’s activity profile to several templates representing distinct temporal features of glucose dynamics, and classified cells based on the best goodness of fit (Fig. 4a and Methods). Based on this classification, the majority of HONs (98%, 895 of 913 cells) responded to blood glucose dynamics, with only 2% of cells not fitting any of the templates (Fig. 4b,d). Most of the glucose-responding HONs were glucose inhibited (64%, of which 31% were glucose-derivative-inhibited cells and 33% were glucose-proportional-inhibited cells), but we also detected other functional subclasses (glucose-derivative-activated cells, 11%; glucose-proportional-activated cells, 25%) (Fig. 4b,d). The summed/population-level response (Figs. 1 and 3c) resembled that of glucose-derivative-inhibited cells (idG cells in Fig. 4b), presumably because of the small response amplitudes of glucose-derivative-activated cells (dG cells in Fig. 4b) and the relatively similar response amplitudes of glucose-proportional-inhibited and glucose-proportional-activated cells (iG and G cells in Fig. 4b). We could also recreate the HON population response to glucose infusion by multiplying the glucose templates by the relative HON-subgroup prevalence (Extended Data Fig. 4a). The HON subsets were largely intermingled within the recording volume (Fig. 4e).

We next asked how other information is distributed across these glucose-classified HONs. On faster timescales than the glucose dynamics analyzed above, individual HONs displayed heterogeneous temporal coupling to locomotion, with some neurons positively correlated, some negatively correlated and some noncorrelated with running^15^. We reexamined our new data at a high temporal resolution from this perspective of running, which we recorded concurrently with neural activity in all the two-photon experiments. When we combined the insights about the slow glucose responses and the fast running responses, we found that most of the running-correlated HONs were glucose inhibited, whereas most of the HONs that were negatively correlated with running were glucose excited (Fig. 4d, right). In agreement with this, glucose-inhibited cell subpopulations had larger proportions of cells positively correlated with running than the other classes of HONs (Extended Data Fig. 4b).

Overall, these data indicate that, while there are distinct subpopulations of HONs in terms of correlations with blood glucose dynamics or running, the bulk of HONs are glucose inhibited (proportional-tracking iG and derivative-tracking idG cells in Fig. 4b), and most of the individual HONs that are positively correlated with running are glucose inhibited.

### Role of HONs in glucose-evoked locomotor suppression

**Fig. 5:**
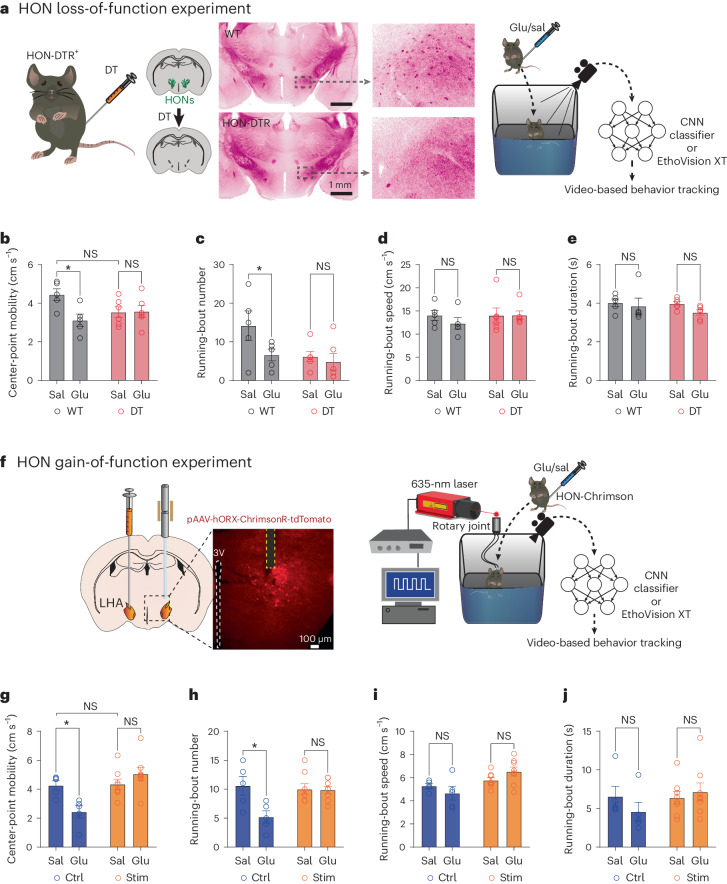
HONs and glucose-evoked behavior. **a**, HON-ablation strategy (left), histology (middle; similar data were obtained in *n* = 12 mice) and behavior assessment schematic (right). CNN, convolutional neural network; sal, saline. **b**, Mobility. Two-way RM ANOVA: treatment × group *F*(1, 9) = 5.4, *P* = 0.04; WT: saline versus glucose, *P* = 0.03; DT: saline versus glucose, *P* = 0.995; *n* = 5 WT mice, *n* = 6 DT mice. **c**, Running-bout number. Two-way RM ANOVA: treatment *F*(1, 9) = 6.933, *P* = 0.03; WT: saline versus glucose, *P* = 0.03; DT: saline versus glucose, *P* = 0.82; *n* = 5 WT mice, *n* = 6 DT mice. **d**, Running-bout speed. Two-way RM ANOVA: treatment × group *F*(1, 9) = 0.71, *P* = 0.42; *n* = 5 WT mice, *n* = 6 DT mice. **e**, Running-bout duration. Two-way RM ANOVA: treatment × group *F*(1, 9) = 0.31, *P* = 0.59; *n* = 5 WT mice, *n* = 6 DT mice. **f**, HON-activation strategy (left), histology (middle; similar images were obtained in each of *n* = 5 mice) and behavior assessment schematic (right). **g**, Mobility. Two-way RM ANOVA: treatment × group *F*(1, 11) = 13.78, *P* = 0.003; control (Ctrl): saline versus glucose, *P* = 0.01; stimulated (Stim): saline versus glucose, *P* = 0.23; *n* = 5 control mice, *n* = 8 Chrimson mice. **h**, Running-bout number. Two-way RM ANOVA: treatment × group *F*(1, 11) = 5.29, *P* = 0.04; control: saline versus glucose, *P* = 0.02; stimulated: saline versus glucose, *P* = 0.9953; *n* = 5 control mice, *n* = 8 Chrimson mice. **i**, Running-bout speed. Two-way RM ANOVA: treatment × group *F*(1, 11) = 3.57, *P* = 0.09; *n* = 5 control mice, *n* = 8 Chrimson mice. **j**, Running-bout duration. Two-way RM ANOVA: treatment × group *F*(1, 11) = 1.38, *P* = 0.26; *n* = 5 control mice, *n* = 8 Chrimson mice. All group data are presented as means and s.e.m. *P < 0.05; *P* > 0.05 (NS). Source data

Finally, we considered the possibility that blood glucose governs behavior and HONs are involved in this process. Locomotion is stimulated by bulk HON activation^15^. This suggests that locomotion may be suppressed by HON inhibition, such as that evoked by glucose. Thus, we hypothesized that glucose suppresses locomotion, and this suppression is sensitive to HON-selective perturbations (Fig. 5a,f).

**Extended Data Fig. 5:**
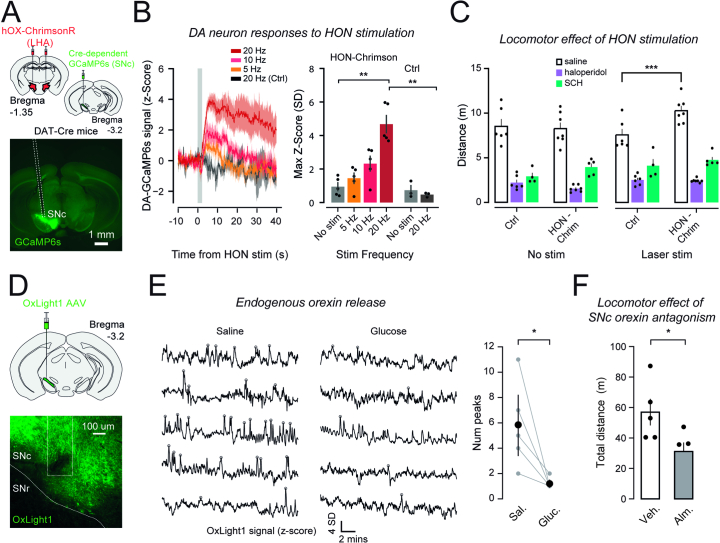
Probing a pathway for HON regulation of locomotion. Related to Fig. 5. **(A)** Schematic drawings of AAV injections for HON optostimulation combined with SNc DA neuron photometry (top), and representative histology (bottom, fiber placement shown as dashed rectangle). Similar histology was obtained in n = 5 mice. **(B)** DA neuron responses to HON optostimulation. Left, SNc GCaMP fluorescence responses to HON optostimulation (grey bar). n = 5 HON-ChrimsonR mice, and n = 3 control (ctrl) mice. Right, Peak fluorescence (95th percentile) in a 20 second window after stimulation onset; paired t-test t_4_ = 6.271 p = 0.007. Right, Comparison of 20 Hz stimulation in Chrimson vs control (Ctrl) mice; unpaired t-test t_6_ = 6.131, p = 0.002 (n = 5 HON-ChrimsonR mice, and n = 3 control mice). Multiple comparisons were Bonferroni adjusted. **(C)** Effect of DA antagonist drugs (haloperidol, SCH23390) or saline on locomotor responses to HON optostimulation. Mice were placed in an open field to assess movement for 5 minutes either without (no stim) or with LH laser stimulation (Laser stim, 40 Hz). Anova, p = 0.0078, F (1, 29) = 8.178, with Šídák’s posthoc test, control vs chrimson saline, p = 0.0004; other effects were not significant (Laser stim, control vs Chrimson haloperidol, p = 0.9992; control vs chrimson SCH, p = 0.7799), n = 6 mice (control saline and haloperidol), n = 4 mice (control SCH), n = 7 mice (Chrim saline and haloperidol), n = 5 mice (Chrim SCH). **(D)** Schematic drawing of AAV injection for SNc orexin level photometry recording (top), and representative histology (bottom, fiber placement shown as dashed rectangle). Similar histology was obtained in n = 5 mice. **(E)** Effect of glucose on SNc orexin levels. Left, each row is a trial from a different mouse. Mice were injected with either saline or glucose (2 g/kg, given 3 min before the start of the traces). Open circles depict identified peaks. Right, Number of OxLight1 fluorescence peaks detected during 20 min period after injection of either saline (white box) or glucose (gray box). Paired t-test, p < 0.02 (n = 5 mice). **(F)** Effect of SNc infusion of orexin antagonist (alm) on locomotion. Five minutes after infusion of either almorexant or vehicle mice were placed in an open field arena for 20 min during which their distance travelled was recorded. Paired t-test, p = 0.0473 (n = 5 mice). All group data shown as means and s.e.m. Source data

In support of the first part of this hypothesis, we observed that glucose suppressed locomotion (Fig. 5b, dataset labeled ‘WT’) in the same epoch in which bulk HON inhibition occurs (Figs. 1 and 3). This involved a reduced number of running bouts, but no change in bout speed or duration (Fig. 5c–e, datasets labeled ‘WT’). To probe how HON inhibition may suppress locomotion, we considered the substantia nigra pars compacta (SNc), a brain region with a high density of HON axons^18^. The SNc contains dopamine (DA) neurons that promote locomotion^19^, are activated by HON firing (implied by ref. ^20^ and confirmed in vivo in Extended Data Fig. 5a,b) and are thought to release DA onto DA receptors critical for HON-evoked locomotion^21,22^ (Extended Data Fig. 5c). Using SNc-targeted photometry of the orexin/hypocretin peptide sensor OxLight1 (ref. ^23^), we found that glucose reduced endogenous orexin release in the SNc (Extended Data Fig. 5d,e). This would reduce endogenous orexin receptor activation. To test whether reducing endogenous orexin receptor activation is sufficient to suppress locomotion, we infused the orexin receptor antagonist almorexant^24^ locally into the SNc. This suppressed locomotion (Extended Data Fig. 5f). Together, these results suggest that glucose reduces pro-locomotor orexin signals in the SNc.

To test the second part of our hypothesis, we designed two different but complementary experiments probing the role of HONs in glucose-evoked locomotor suppression. The loss-of-function experiment examined the suppression of locomotion when HONs were selectively ablated in the HON-diphtheria toxin (DT) receptor (DTR) model^25^ (Fig. 5a–e). The gain-of-function experiment examined the suppression of locomotion when HON activity was artificially maintained at a high level by HON-selective optostimulation (Fig. 5f–j). Each of these experiments has distinct potential confounds (for example, baseline shifts and chronic effects), but combining the evidence from the two experiments reduces interpretation difficulties arising from these confounds.

**Extended Data Fig. 6:**
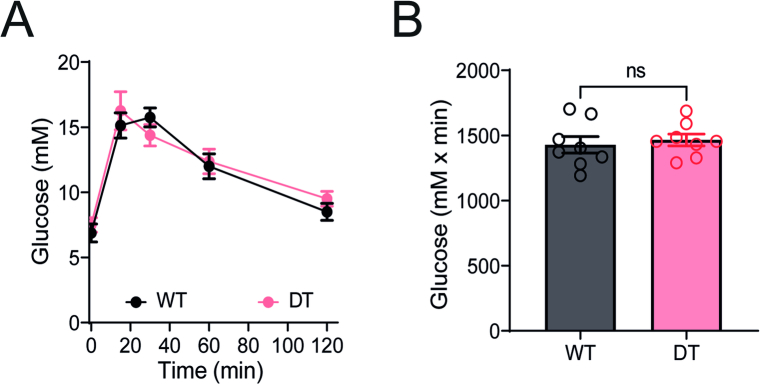
Glucose tolerance test (GTT) in wild-type and HON-ablated mice. Related to Fig. 5. **(A)** GTT timeline. (**B**) GTT area under the curve. Unpaired t-test p = 0.56, t = 0.59, df=14, n = 8 WT and 8 DT mice. Data presented as means and s.e.m. of n = 8 WT and 8 DT mice. Source data

In the HON-ablated mice, glucose infusions increased blood glucose as normal (Extended Data Fig. 6a,b) but no longer suppressed locomotion (Fig. 5b–e, dataset labeled ‘DT’). As expected^15^, there was a notable (but not statistically significant) reduction in baseline locomotion in HON-ablated mice (saline DT group versus WT groups; Fig. 5b,c). Thus, it was important to confirm this observation in a different experiment without this downward trend. This was accomplished in the HON optostimulation experiment, which also prevented glucose-evoked locomotor suppression (Fig. 5g–j). Together, these two complementary experiments indicate that intact HON activity dynamics are important for normal motor responses to glucose.

## Discussion

In sensors, the question of whether an input is simply relayed (proportional tracking) or tracked in a more selective and specialized way (derivative or integral tracking) has great functional implications^1,26^. This is because proportional, integral and/or derivative tracking modes enable reactions based on the present, past and future of an input, respectively^26,27^. Identifying the temporal nature of input perception thus reveals fundamental information about the overall operational logic of a sensory system. This has been of broad interest for glucose-tracking systems and the design of artificial glucose trackers in clinically relevant applications, such as the artificial pancreas^28–37^. By identifying derivative and proportional tracking of blood glucose in HONs, our study directly visualizes this fundamental aspect of HON operation in the living brain.

In behaving mice, we found that HONs transmitted multiple temporal features of blood glucose dynamics. At the population level, the dominant feature transmitted by the low-frequency HON response was the glucose derivative (Fig. 2). At the single-cell level, HONs operated as multiplex transmitters, in which low-frequency glucose modulation typically coexisted with high-frequency locomotion modulation (Fig. 4d). Multiplexing increases communication efficiency by sending diverse information streams through one communication channel^38^, in our case, one HON. This reduces the number of neurons required to transmit the diverse information. This is thought to be important because neurons are energetically expensive^39^, and physical space to accommodate them in the brain is limited, thus placing constraints on information processing. Therefore, the multiplexing of diverse information within one neuron may allow for the more efficient use of the limited brain resources for neural coding^40,41^. We speculate that, when an animal is experiencing a wide variety of slowly and rapidly changing stimuli, as typically occurs in the wild, demultiplexer circuits downstream of HONs may be able to extract information about the distinct stimuli from the superimposed slow and fast HON modulation.

Anticipatory algorithms based on associative cues and learning are increasingly documented in the hypothalamus^42–45^. In addition to these cue-based algorithms, there is a fundamentally different type of anticipatory algorithm that is cue independent and instead involves emitting control signals based on the temporal derivative of an input^26^. This derivative-based algorithm is well established as a simple and effective way of creating rapid, anticipatory-like signals in practical and theoretical engineering^26,27^. Our data suggest that this algorithm may also operate in the hypothalamus. If glucose is viewed as an input, the glucose derivative-based HON population signal (Fig. 1) closely resembles derivative-based anticipatory control signals. Control is more effective when it uses the derivative signals because their anticipatory-like dynamics preempt big deviations from a set point^26^. We propose that understanding and mimicking the natural mathematics of the brain’s glucose-sensing strategies may improve the performance of clinically relevant glucose-sensing artificial organs^28,29,36^.

Our data indicate that HONs have an essential role in glucose-evoked locomotor suppression (Fig. 5). This may have an evolutionary advantage by suppressing moving away from a glucose-containing food source, thus facilitating additional consumption and/or energy storage^46–48^. The essential role of HONs in this locomotor suppression is supported by two independent—but interpretationally complementary—observations: the loss of suppression upon selective HON ablation and the overriding of suppression by selective HON hyperstimulation (Fig. 5). Physiologically, HONs are activated by diverse salient signals, such as those indicative of stress or a reward^49^. In future work, it would be important to test whether the suppression of locomotion by glucose may be overridden by salient signals that activate HONs, to permit behavioral flexibility.

Our findings open additional directions for further study. Downstream of HONs, it could be elucidated whether HON subsets with distinct glucose responses engage distinct or overlapping decoders and effectors. While the midbrain may be one such effector (Extended Data Fig. 5), other HON-innervated areas^18^ are likely to contribute. In particular, areas involved in metabolic control deserve further investigation, as the apparently normal glucose tolerance of HON-ablated mice does not rule out potential HON effects on multiple other aspects of metabolism. Upstream of HONs, other glucose-interpreting systems may provide inputs to HONs, as the in vivo diversity of HON glucose representations (Fig. 4) is absent in isolated HONs in vitro^6,7^. While indirect HON glucose sensing through such inputs seems likely, we note that the fact that peak HON responses precede peak blood glucose increases does not prove indirect sensing, as it would be equally explainable by direct derivative sensing. Thus, it remains to be experimentally established—for example, by inactivating putative upstream sensors—whether the in vivo HON glucose responses arise from inputs from other glucose sensors^50^ or through some combination of direct^6,7^ and indirect^50^ sensing. It is also interesting that the relationship between HON population activity and glucose displayed hysteresis (Fig. 1h). Hysteresis could have many underlying causes^51^, including bistable biological switches and feedback loops^52^. It would be interesting to test whether such operations exist in the broader HON network and whether they implement the glucose derivative tracking. Finally, on a whole-body level, it remains to be determined how HON glucose sensing works together with other hypothalamic sensors of blood glucose^53^, as well as with multiple other blood and interstitial glucose sensors, to produce an integrated response to multicompartment glucose dynamics^54,55^.

Together with the transcriptomic and projection complexity of the HONs, the temporal dimension revealed here may aid downstream circuits in efficiently interpreting the wide range of information transmitted by HONs. Deeper knowledge of how precisely defined neural classes and connections turn blood glucose into adaptive responses will facilitate insights into the impact of glucose dynamics on health and disease.

## Methods

### Experimental subjects

All animal experiments followed the UK Home Office regulations or the Swiss Federal Food Safety and Veterinary Office Welfare Ordinance (TSchV 455.1, approved by the Zurich Cantonal Veterinary Office). Adult male C57BL/6 mice were studied; thus, whether conclusions apply to female mice remains to be determined. For HON-deletion experiments (Fig. 5 and Extended Data Fig. 6), we used the already validated orexin-DTR mice^25,46^; the confirmatory histology shown in Fig. 5a was performed as in ref. ^46^. For SNc DA neuron recordings (Extended Data Fig. 5a,b), we used DA transporter-Cre transgenic mice (The Jackson Laboratory 020080, *Slc6a3*^*tm1(cre)Xz*^/J). The animals were housed in a reversed light–dark cycle (lights off at 7:00 a.m.), and all experiments were performed during the dark phase. The animals had ad libitum access to food (3430 Maintenance Standard diet, Kliba Nafag) and water unless stated otherwise. Known effect sizes and variations were used for power calculations and the determination of the required number of animals, where possible, to maximize the chances of meaningful results without using excessive numbers of experimental animals. Studies were repeated in at least two independent cohorts and used a semirandomized crossover design.

### Surgeries and viral vectors

HON activity measurement or optogenetic control was achieved using an orexin promoter (hORX)-driven GCaMP6s sensor or the excitatory optogenetic actuator ChrimsonR, respectively; the specificities of these constructs for HONs have been validated by histological analyses^15,23,25^. Briefly, the GCaMP6s calcium indicator was delivered using AAV1-hORX-GCaMP6s.hGH (10^13^–10^14^ genome copies (GC) per ml, Vigene Biosciences). For fiber photometry recordings of HONs, the hORX-GCaMP6s adeno-associated virus (AAV) was stereotaxically injected into the LH bilaterally (anteroposterior (AP), −1.35 mm; mediolateral (ML), ±0.90 mm; dorsoventral (DV), −5.70, −5.40 and −5.10 mm; 70 nl per site), and optic fiber cannulae (200-μm diameter, 0.39-numerical-aperture (NA) fiber with 1.25-mm ceramic ferrule; Thorlabs) were implanted above the LH (AP, −1.35 mm; ML, ±0.90 mm; DV, −5.00 mm). For fiber photometry recordings of SNc DA cells (Extended Data Fig. 5a,b), Cre-dependent GCaMP AAV (pAAV.CAG.Flex.GCaMP6s.WPRE.SV40, 1.7 × 10^13^ GC per ml; 1:3 dilution in sterile PBS) was stereotaxically injected into the SNc unilaterally (AP, −3.2 mm; ML, ±1.4 mm; DV, −4.2 mm; 200 nl per site at 1 nl s^−1^), and optic fiber (200-μm diameter, 0.39-NA fiber with 1.25-mm ceramic ferrule; Thorlabs) was implanted above the SNc 0.1 mm above the injection site. For fiber photometry recordings of SNc orexin/hypocretin levels (Extended Data Fig. 5d,e), 300 nl of the OxLight1 sensor AAV (~7 × 10^12^ GC per ml, AAV DJ, UZH Viral Vector Facility) was stereotaxically injected unilaterally into the SNc (the coordinates and subsequent fiber implantation are the same as in the previous sentence). For central infusion of almorexant, a bilateral cannula (RWD Life Science) was introduced into the SNc (Extended Data Fig. 5f). For two-photon imaging, the same GCaMP6s virus and coordinates were used as for fiber photometry; however, surgery was performed only on the left hemisphere, and a GRIN lens (0.39-NA, 7.3-mm-long, 0.6-mm-diameter; Inscopix) was slowly (150 μm min^−1^) implanted instead of the cannulae. The implants and a custom-made aluminum head plate were secured to the skull using three skull screws and dental cement (Kemdent dental cement and C&B Metabond (Parkell)). For optogenetic HON experiments, surgeries were performed in the same way as for the HON photometry studies, but ChrimsonR was delivered using AAV9-hORX-ChrimsonR-mCherry (2 × 10^12^ GC per ml, UZH Viral Vector Facility).

For IG infusion experiments, the surgery was adapted from that performed in rats^46^. A custom-made catheter was implanted by guiding the tubing subcutaneously from the dorsal to the ventral incision. A fixation mesh was then positioned subcutaneously at the level of the scapula blades, and the catheter exited on the animal’s back. To prevent damage, we placed a custom-made cover on the catheter after every experiment. To prevent catheter blockage, we used saline (~0.1 ml) to flush the catheters daily for 5 days after surgery and then every second day until the end of the study. Catheter functionality was confirmed by the presence of backflow after saline IG infusion and terminally by dissection. In the event of catheter blockage, subjects were excluded from further experiments, but data collected before the blockage were included in the analyses.

Glucose and temperature telemeters (DSI) were implanted following the manufacturer’s instructions. The glucose sensor entered the systemic blood system through the left carotid artery, with ~1.5 mm of the sensor protruding into the aorta. All surgeries were performed under aseptic conditions. The animals received isoflurane anesthesia and operative and postoperative analgesia.

### Fiber photometry

For fiber photometry experiments (Figs. 1 and 2 and Extended Data Fig. 5b,e), we used a custom camera-based photometry system (built with the assistance of D. Elgar (Custom and open-source systems for neuroscience (COSYS)) and based on ref. ^56^). Alternating illumination from two excitation LEDs (405 and 465 nm at 20 Hz each, average power of 100 μW at the implant fiber tip) was used to record LH HON-GCaMP6s emission fluorescence bilaterally in one to three animals simultaneously. The emission generated by the 405-nm LED was used as a control for movement artifacts, the effects of which were further minimized by recording in habituated, head-fixed animals (Fig. 1a). GCaMP6s bleaching, fiber illumination and expression variability were accounted for by detrending and normalizing each trace as follows: (1) the local minima of each trace were found (‘convhull’ function in MATLAB); (2) a least-squares triple exponential was fit through the convex hull; (3) each trace was detrended by subtracting and dividing by its minima fit; and (4) each trace was *z*-score normalized based on its 20-min preinfusion s.d. and mean.

### Two-photon imaging and data analysis

We performed volumetric two-photon imaging using GRIN lenses^15^. Excitation of GCaMP6s was achieved with a femtosecond-pulsed mode-locked Ti:sapphire laser (Spectra-physics Mai Tai HP Deepsee 2) at 950 nm. The emission fluorescence was imaged using a resonant/galvanometer-scan-head two-photon microscope (Independent NeuroScience Services) equipped with a 20× (0.45-NA, Olympus) air-IR objective, a custom electrotunable lens and a 510/80-nm band-pass emission filter. A volume of 512 × 512 pixels × 6 planes was recorded at 5.1 volumes per second using custom LabVIEW software. Resultant image stacks were processed in FIJI, MATLAB and Python software programs, as follows: (1) the imaged volume was split into separate planes (the lens-transition plane was discarded from further analysis); (2) 2 × 2 binning and TurboReg (precise, rigid) motion correction were applied; (3) cell outlines were manually drawn and labeled as ROIs; (4) ROI maps were applied across sessions within the same animal and adjusted; (5) ROIs that corresponded to the same cell in a plane or across neighboring planes were identified (in at least two independent experimental sessions, >5% ROI overlap of 3-pixel expanded contours, >90% cross-correlation coefficient and <2 s lag) and joined in further analysis; (6) HON cell activity was aligned based on saline/glucose infusion timing, and same-condition recordings were averaged for each cell; (7) HONs were assigned to glucose-response classes by fitting their activity profile to transformations of glucose dynamics (inverted-sign derivative, derivative, proportional and inversely proportional); and (8) classified cells were anatomically mapped.

For the classification of HON activity to transformed glucose dynamics, templates were constructed using the average blood glucose trace following an IP injection of 2 g kg^−1^ glucose from mice that were not used in the classification. The mean trace was smoothed using a 1.5-min-window moving mean before relevant transformations were applied. If the transformation was a derivative, the template was smoothed again after differentiation using the same filter parameters. Cell activity traces were extracted and aligned to the first 20 min of the template trace following the IP injection of glucose. By calculating the Pearson’s correlation coefficient of the extracted traces with each of these templates, we assigned cells to the response class with the maximum Pearson’s correlation coefficient. A two-sided *P* value was calculated alongside each correlation (SciPy library). Using the Bonferroni adjustment for multiple comparisons, we classified responses that had no *P* value with any template less than *α* = 0.001/4 as belonging to a fifth (‘no-response’) category.

For the classification of individual HONs with regard to their correlation with running events at fast timescales^15^, we used the 5-Hz data from whole sessions. Positively correlated (*P* < 0.05, *ρ* > 0.01), negatively correlated (*P* < 0.05, *ρ* < −0.01) and noncorrelated (*P* > 0.05 or *ρ* between −0.01 and 0.01) cells were identified using Spearman’s correlation.

### Concurrent monitoring of glucose, metabolic parameters and locomotion

Blood glucose concentration and flank body temperature were measured and preprocessed with the HD-XG telemetry system (DSI). Metabolic measurements were obtained by recording continuous gas exchange in a custom enclosure by using an adapted Field Metabolic System (Sable Systems International). The running of head-fixed animals was measured on a wheel using an optical encoder (Honeywell, 128 ppr 300 rpm Axial). Encoder state changes were recorded using a master MATLAB code running photometry or LabVIEW programs synced with the two-photon microscope. Metabolic data were preprocessed in ExpeData software, *z*-transformed to account for sensor lag and exported for analysis in MATLAB. Respiratory energy expenditure was calculated using Weir’s formula: energy expenditure (kcal min^−1^) = (3.94 × VO_2_ (l min^−1^)) + (1.1 × VCO_2_ (l min^−1^)). All acquisition systems were synced using digital signals to the MATLAB code running photometry or to the LabVIEW two-photon imaging program. All data were exported and further processed in MATLAB. Each of the simultaneously measured time series was resampled to achieve the same acquisition rate of 1 Hz. For visual clarity, glucose and photometry data in Figs. 1 and 2 were smoothed with a 10-min moving mean and downsampled to 20 s. To generate hysteresis plots of HON activity versus blood glucose from diverse infused glucose parameters in Fig. 1, temporal boundaries of the blood glucose transient were identified, and 50 equally spaced points were taken to plot the HON signal versus the blood glucose level (Fig. 1h) or the blood glucose derivative (Fig. 1k). Classical glucose tolerance tests (Extended Data Fig. 6a,b) were performed as in our previous work^25^.

### Glucose doses

A critical feature of our experimental design is that, because of the rapid physiological counter-regulation of glucose in the body, in all our analyses of HON population responses to glucose, we used actual measured blood glucose values rather than the administered glucose doses. In this way, we assessed the effects on neural activity of a large range of blood glucose concentrations (baseline range, 3.5–11.1 mM (mean, 7.3 ± 0.17 mM); glucose peak range, 11.1–34.9 mM (mean, 24 ± 0.9 mM)) and rates of change (range, 0.12–1.45 mM min^−1^; mean of maximum rate of change, 0.56 ± 0.03 mM min^−1^). These glucose parameters are comparable to physiological variations of blood glucose in mice^57–59^. To achieve this range of glucose variations, we varied our glucose infusions in both concentration and route of administration (IG or IP). Specifically, for IG infusions, we used a range of concentrations (0.08, 0.12, 0.146, 0.24 and 0.45 g ml^−1^), infusion rates (50, 66.7, 90 and 180 μl min^−1^) and volumes (<0.45 ml for fast infusions of 180 μl min^−1^ and <0.9 ml for the other rates). For IP glucose infusions, a dose of 2 g kg^−1^ was achieved by injecting 100–150 μl. All infusion parameters were used to generate the data analyzed in Fig. 1. An IG dose of 0.24 mg ml^−1^ (delivered at a rate of 90 μl min^−1^ over 10 min) was used in Fig. 2; in Extended Data Fig. 2, the findings are confirmed for a wider range of infusion parameters (as indicated in the legend of Extended Data Fig. 2). An IP dose of 2 g kg^−1^ was used in Figs. 3–5. Note that the administration routes and doses are provided here for the sake of completeness, as our study relates blood glucose (directly measured rather than inferred from injected doses) to HON activity, and the relationship between blood glucose and HON activity is similar across different methods of glucose injection (Extended Data Fig. 1, IP and IG compared in the figure legend).

### Encoding model

To determine the relative contributions of various behavioral and metabolic variables to HON responses, we used a generalized linear model approach based on ref. ^17^. For this model, HON population activity was used as the response variable, whereas the predictor variables were running, blood glucose, body temperature, VO_2_, VCO_2_ and their derivatives with respect to time (Fig. 2h). All variables were downsampled to 1 min to equalize their sampling rates and focus on the slow dynamics, Savitzky–Golay filtered (first-order, five-sample window) and normalized by *z* scoring. Resultant data were fit using the ‘glmfit’ function in MATLAB by bootstrapping 1/4 experiment-duration chunks randomly over 2,000 iterations. Here, 70% of the data were used for training, and 30% were used for validation on untrained data. Nonbootstrapping methods, such as leave-one-out cross-validation, were also used to confirm the findings. Examination of the correlation coefficient matrix of predictor pairs confirmed low collinearity (correlations ≪ 0.8)^60^, as expected, because, in addition to the temporal variations in blood glucose concentration, CO_2_ production and O_2_ consumption depend on other dynamic variables—for example, the intensities of aerobic and anaerobic intracellular metabolisms and glycogen metabolism. This presence of multiple variables that display distinct temporal patterns provides further justification for the multivariate modeling approach, which attempts to disentangle this complex situation by looking at the temporal covariability of the different metabolic parameters as predictors of the temporal activity patterns of HONs. For each fitting iteration, partial models based on the same training data, but without a single independent variable, were generated. Then, a coefficient of determination (*R*^2^) was calculated for full and partial models on the validation data (either on the 30% of bootstrapped data or the left-out experiment). We determined the relative contribution of a given predictor to HON activity dynamics by comparing how much the encoding model performance has declined without a given variable—by comparing the *R*^2^ of the partial model to the *R*^2^ of the full model (Fig. 2i). Negative relative contributions were set to zero, following the method described in ref. ^17^.

### Behavioral analysis

HON-ablated mice and their respective controls were produced by injecting DT (Sigma D0564, 1 mg ml^−1^, 0.1 ml) through the IP route into both DTR^+^ and DTR^−^ mice^25^. A constitutively active HON state was produced by optogenetic LH stimulation of HON-Chrimson mice, achieved by tonic 10-Hz, 5-ms pulses of 635-nm laser. In both cases, HON-manipulated and control mice were injected with either glucose or saline (in a randomized crossover fashion over two consecutive days) and placed in an open-field arena (40 × 40 cm). Behavior was scored using video analysis of the first 10 min by EthoVision XT (Noldus) and a custom-made machine-learning classifier^46^. The machine-learning classifier was trained on ~1,200 labeled examples to identify five separate behaviors: grooming, rearing, resting, running, and turning or sniffing. More than 300 additional labeled examples were used to train a behavioral classifier for optogenetic-cable-tethered mice. Separate running bouts were defined as occurrences of movement above a threshold (18 cm s^−1^ for untethered mice or 10 cm s^−1^ for tethered mice) for at least 1 s per bout, separated by more than 2 s. Similarly, when machine learning was used to identify forward locomotion directly, separate running bouts were identified when spaced by at least 2 s.

### Data analysis and statistics

Raw data were processed in MATLAB. Statistical analysis was done in GraphPad Prism 9.0, MATLAB or Python. For data analysis, the researcher was blinded to group identity; behavioral quantification (pupil size) was performed in an unbiased, automated way using EthoVision or custom behavioral classifiers. Key comparisons between saline and glucose, as well as cell classification analyses, were performed on raw, nonsmoothed data. Sample size, statistical tests used and their results are indicated in the figures, figure legends and/or descriptions in the text. No statistical methods were used to predetermine sample sizes, but our sample sizes are similar to those reported in previous publications^15,46^. Statistical comparisons were performed on nonfiltered data, but some traces were filtered for visual purposes, as indicated. Statistical analysis was based on settings recommended by GraphPad Prism 9.0. *P* values of <0.05 were considered significant. Where relevant for testing, data distribution was assumed to be normal, but this was not formally tested. Where significance is presented, *P* values are as follows: *P < 0.05, **P < 0.01, ***P < 0.001 and ****P < 0.0001; NS, *P* > 0.05. Outliers that failed a ROUT 1% test were removed. Data are presented as means and s.e.m. unless stated otherwise. All *t* tests were two-tailed.

### Ethics declaration

The study was carried out in accordance with the inclusion and ethics statement of the Nature Publishing Group.

### Reporting summary

Further information on research design is available in the Nature Portfolio Reporting Summary linked to this article.

## Online content

Any methods, additional references, Nature Portfolio reporting summaries, source data, extended data, supplementary information, acknowledgements, peer review information; details of author contributions and competing interests; and statements of data and code availability are available at 10.1038/s41593-024-01648-w.

### Supplementary information

Reporting Summary

### Source data

Source Data Fig. 1 Statistical source data.

Source Data Fig. 2 Statistical source data.

Source Data Fig. 3 Statistical source data.

Source Data Fig. 5 Statistical source data.

Source Data Extended Data Fig. 1 Statistical source data.

Source Data Extended Data Fig. 5 Statistical source data.

Source Data Extended Data Fig. 6 Statistical source data.

## Data Availability

Source data for the main figures can be found at 10.7910/DVN/KCKNG3. Source data are provided with this paper.

## Code availability

Custom codes used in the article can be found at https://github.com/Pauvis/HON-Gluocse.git.
